# Follow up of MRI bone marrow edema in the treated diabetic Charcot foot – a review of patient charts

**DOI:** 10.1080/2000625X.2018.1466611

**Published:** 2018-04-26

**Authors:** Ernst-A. Chantelau, Sofia Antoniou, Brigitte Zweck, Patrick Haage

**Affiliations:** a Department of Endocrinology and Diabetology, Diabetic Foot Clinic, Heinrich-Heine-University Düsseldorf, Düsseldorf, Germany; b Department of Diagnostic and Interventional Radiology, HELIOS University Hospital Wuppertal, University Witten/Herdecke, Wuppertal, Germany

**Keywords:** Diabetes mellitus, polyneuropathy, neuro-osteoarthropathy, fracture healing, neuro-arthropathy, diabetic foot syndrome

## Abstract

**Background**: Ill-defined areas of water-like signal on bone magnetic resonance imaging (MRI), characterized as bone marrow edema or edema-equivalent signal-changes (EESC), is a hallmark of active-stage pedal neuro-osteoarthropathy (Charcot foot) in painless diabetic neuropathy, and is accompanied by local soft-tissue edema and hyperthermia. The longitudinal effects on EESC of treating the foot in a walking cast were elucidated by reviewing consecutive cases of a diabetic foot clinic.

**Study design**: Retrospective observational study, chart review

**Material and methods**: Cases with active-stage Charcot foot were considered, in whom written reports on baseline and follow-up MRI studies were available for assessment. Only cases without concomitant infection or skin ulcer were chosen, in whom both was documented, onset of symptomatic foot swelling and patient compliance with cast treatment.

**Results**: From 1994 to 2017, 45 consecutive cases in 37 patients were retrieved, with 95 MRI follow-up studies (1–6 per case, average interval between studies 13 weeks). Decreasing EESC was documented in 66/95 (69%) follow-up studies. However, 29/95 (31%) studies revealed temporarily increasing, migrating or stagnating EESC.

**Conclusion**: EESC on MRI disappear in response to prolonged offloading and immobilizing treatment; however, physiologic as well as pathologic fluctuations of posttraumatic EESC have to be considered when interpreting the MR images. Conventional MRI is useful for surveillance of active-stage Charcot foot recovery.

## Abbreviations

EESC edema-equivalent signal-changes

MRI magnetic resonance imaging

FUS follow-up study

FS fat-saturated

PD proton density

SE spin echo

SPIR spectral presaturation with inversion recovery

STIR short tau inversion recovery

T1w T1 weighted

T2w T2 weighted

TCC total contact cast

TSE turbo spin echo

## Introduction

Magnetic resonance imaging (MRI) in recent years has become state of the art for diagnosing the active-stage Charcot foot in patients lacking pedal nociception (neuro-osteoarthropathy). The potential of MRI for monitoring the treatment response, however, as yet has not received much attention [1–5]. For this purpose, clinicians still prefer symptoms and plain x-ray, which is insufficient, because Charcot patients cannot communicate pain-mediated corollaries of treatment, and x-ray cannot disclose subtle acute injuries of the foot skeleton [6].

Early detection and grading of bone injuries, as required for early and appropriate injury management, is available with use of MRI [7]. Hallmark is the so called bone marrow edema like signal (henceforth called edema-equivalent signal-changes EESC), characterized by ill-defined areas of high (water-isointense) signal intensity on T2w and STIR sequences in bone, together with similar signals in the adjacent soft tissues [7–10]. EESC may be depicted on T1w images as hypointense signal. Active processes display enhanced contrast media uptake. The entity of EESC is strictly confined to magnet resonance technique. EESC corresponds to tissue vascularity (hyperemia due to fibrovascular infiltrate) at the site of an infectious or traumatic bone injury [8–15], rather than to increased interstitial fluid content (from diffusion by microvascular hyperpermeability), which is more prominent in the adjacent soft tissues. In the active-stage Charcot foot, closed subcortical trabecular microfractures (bone bruise), or cortical macrofractures are typical. While the former is caused by excessive cyclic loading, the latter is mostly caused by a single high-energy impact, but also by unlimited repetitive stress (fatigue fracture). These injuries are symptomatic only by pedal swelling, hyperthermia, and lymphedema [16], while pain is merely absent due to the underlying neuropathy. Since any skeletal healing is impeded by infection, movement and pressure, EESC in the foot will deteriorate unless these are eliminated [17,18]. Hence, in active-stage Charcot foot, immobilization and offloading causes posttraumatic EESC to decline [2–5]. The evolution of this decline was examined by review of sequential MRI studies available from patient charts.

## Materials and methods

**Study design**: retrospective, observational, uncontrolled cohort study, as part of project No. 2560 approved by the local ethical committee.

**Objective**: To assess temporal changes of EESC during immobilizing and offloading treatment of active-stage Charcot foot.

**Setting**: outpatient diabetic foot clinic

**Material and methods**: clinical audit of patient charts

### Inclusion criteria

Cases of active-stage Charcot foot were included, in which serial MRI reports (of at least a baseline and one follow-up MRI study) were available under treatment with immobilization and offloading, irrespective of the state of the treatment. The diagnosis of active-stage diabetic Charcot foot was based on typical clinical and MRI findings [19]. Onset of the condition was assumed, when the key symptom, foot swelling, was first recognized and other pathology like a gout attack had been excluded. Severity at baseline [20] was categorized as grade 0 (defined by normal x-ray and subchondral microcracks or subcortical trabecular microfractures, and/or loss of joint space [21] and grade 1 (cortical fractures). Cases with skin defects or infections were excluded, as were noncompliant patients, and cases with insufficient clinical documentation (e.g. of adherence to treatment, or of objective/obvious symptoms like foot swelling, hyperthermia or deformation).

### MRI reports

According to the institution’s routines, conventional MRI studies of the foot were commissioned from available radiologists’ practices and/or departments of radiology, irrespective of an expertise with the diabetic Charcot foot. MRI reports were mailed to the hospital and collected in the patients’ files. The studies, albeit not standardized in terms of MR imaging protocols and reporting, were nevertheless in keeping with current clinical requirements in Germany. According to the reports on file, in each of the MRI studies two or more of the following conventional pulse sequences had been performed: T1 weighted sequences (T1-SE; fat-saturated T1FS and T1SPIR, STIR (short tau inversion recovery)), T2 weighted sequences (T2TSE), proton density (PD) weighted sequences with/without fat saturation [20]. Sagittal, coronal and axial planes were always reported, whereas magnetic field strength and brand of the MRI device was nearly never mentioned. While EESC regularly was described in terms of localization and size, adjacent soft-tissue edema was addressed only occasionally. The style of reporting was descriptive and subjective, in accordance with common clinical usage [13,22,23]. For the purpose of the investigation, anatomical and MR signal details indicating BME lesions were abstracted from the MRI reports on file and registered in a custom made standard form (see below). EESC lesions were counted.

### MRI report reliability

A small random sample of the patients (*n* = 6) had received the original MRI scans on DVD-ROM from the radiologists’ practices. These DVDs (*n* = 30) were re-assessed by an external reviewer unaware of the MRI reports on file (PH, specializing in clinical radiology). To this end, the same standard form (see below) as for extracting the reports on file was used. EESC lesions were counted; agreement between the reviewer counts and the counts extracted from the reports on file was taken as proof of their accuracy.

### Clinical routines

Clinical routines were not strictly standardized or controlled, and changed over time. Clinical findings (e.g. hyperthermia, swelling, deformity, joint dysfunction, skin abnormality) were not measured objectively, but rated semi-quantitatively at best (by bi-manual comparative palpation, and by inspection). Patient history (e.g. concerning the date of symptom onset, treatment compliance, etc.) relied on self-reporting. Treatment schedule and timing of the MRI follow-up studies (FUS) were optional, at the discretion of the physicians in charge of the foot clinic. MRI studies could be commissioned for the following reasons: (a) for establishing the diagnosis of active-stage Charcot foot, (b) for monitoring of the response to the treatment, (c) for monitoring the tolerance to reloading. In addition, plain x-rays could be deliberately commissioned by the physicians in charge. Treatment in general was initiated (with few exceptions), when the diagnosis of active-stage Charcot foot was established, i.e. at the time of the baseline (first) MRI study. Treatment consisted of permanent (except for bed-rest at night) and prolonged placing of the affected limb in a bi-valved removable total contact walking cast (TCC [24,25]). MRI follow up in most cases was stopped before complete disappearance of EESC. The decision to cease TCC treatment was based on clinical judgment aided by MRI and/or x-ray follow-up findings [25].

### Evaluation

A standard form comprising every single foot bone from tibia to toes was designed for reviewing the MRI study reports (and for re-assessing MRI scans, see above), consistent with previous authors [26]. The form defined 24 anatomic locations of possible EESC lesions, comprising entire bones as well as bone segments. Compact and cancellous bone was assessed separately, as bone healing differs essentially between cortical and cancellous fractures [27]. EESC as described in the reports on file were abstracted and entered into the form. EESC lesions were counted by number. Changes in overall EESC between subsequent FUS were estimated semi-quantitatively as ‘less’ (regression), ‘unchanged’, or ‘more’ (progression), and ‘other’, and were synchronized with related clinical features extracted from the files. Soft-tissue involvement was excluded from the analysis, as it had rarely been mentioned in the MRI reports on file.

### Statistics

Statistics were applied for descriptive purposes. Medians and ranges are given, as indicated. Mann–Whitney U test, Chi^2^ test and intraclass correlation coefficient (ICC) were computed, as appropriate. Lin’s concordance correlation coefficient (rho c) was calculated with 95% confidence interval.

## Results

### Cases and patients

A cohort of 45 cases of active-stage Charcot foot was available for analysis, in 37 patients. Four cases were relapses in 3 patients. Four cases were bilateral one was a relapse). The patients’ clinical characteristics are summarized in Table 1.

**Table 1.** Patients’ characteristics.

Persons, n	37
Gender, f/m, *n*	21/16
Age, years. Median (range)	59 (37–81)
Diabetes-type 1/2, *n*	17/19
No diabetes, *n*	1
Co-morbidity:	
Obesity (BMI>30), *n*	10
Kidney-failure (or -transplantation), n	5

BMI: body mass index.

There were 17 cases of active-stage Charcot foot grade 0, diagnosed 4 (1–52) weeks after symptom onset (= onset of foot swelling), and 28 cases grade 1, diagnosed 12 (4–36) weeks after symptom onset. The initial MRI scan had been unclear in 4 cases, which turned out to be grade 1 on follow up.

### MRI reports on file

There were 140 reports in total, concerning 45 baseline and 95 MRI follow-up studies (FUS). The pulse sequences performed (see above) were indicated in 114 of the reports, whereas magnetic field strength was mentioned in only 3 reports (1,0 or 1,5 T). Of 30 MRI studies (6 patients, 7 cases, 30 MRI reports provided by 13 different radiologists), the original scans were reviewed by expert PH. His EESC lesion counts and those extracted from the reports on file were fairly concordant, as indicated by ICC = 0.8598, and Lin’s concordance correlation coefficient rho c = 0.8556 (95% CI 0.7213–0.9279). Further data are provided in Table 2.

**Table 2.** Agreement of the expert’s MRI scan readings (*n* = 30) with the respective MRI reports on file.

Proportions of readings
Confirming principal EESC: 100%
Recording less EESC: 30%
Recording other EESC: 17%
Confirming overall temporal changes of EESC: 100%

MRI: magnetic resonance imaging.

**Figure 1.**
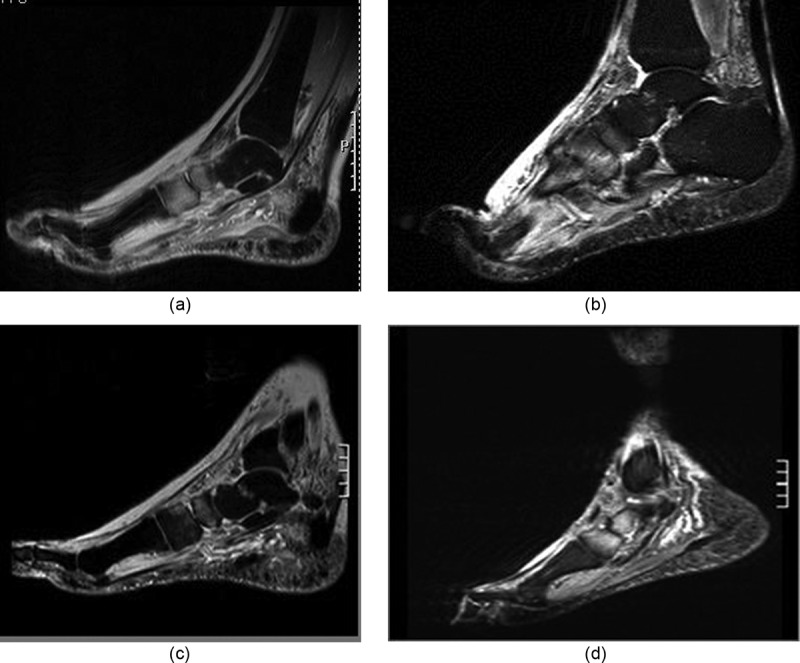
(a) Baseline diagnostic MRI of active-stage Charcot foot grade 0, four weeks after symptom onset. Sagittal STIR sequence showing EESC of tarsal bones (bright appearance), and soft tissue. (b) Same foot as in (a). First follow-up MRI after 6 weeks of unloading and immobilizing. Merely unchanged EESC, as compared to (a). (c) Second follow-up MRI after 11 weeks of treatment. Regression of bone and soft EESC (as compared to (a) and (b)). Unprotected normal weight-bearing was resumed immediately, without weaning. (d) Follow-up MRI after 21 weeks of unprotected re-loading. Relapse of bone EESC, now with tarsal fractures, soft-tissue edema, and collapse of the longitudinal arch, consistent with active-stage Charcot foot grade 1.

From the 45 baseline diagnostic MRI reports on file, a total of 264 bone lesions with EESC were retrieved (on average 6/case). Of these, 29 lesions were located in compact (long) bones or diaphyseal segments, and 243 in cancellous bones or bone segments (cancellous to compact bone ratio being about 8:1). Most of the EESC (n = 204) were located in the midfoot (involving Lisfranc and Chopart joint). For an example, see Figure 1(a)

### MRI follow-up studies (FUS)

There were 2 (1–6) FUS per case: 19 cases had only 1 FUS, while 26 cases had 2 and more FUS (in 11 cases 2 FUS were available, 9 cases had 3 FUS, and 6 cases had 4–6 FUS). Individual FUS were separated by 13 (35–50) weeks on average, see Table 3.

**Table 3.** Time intervals between sequential FUS.

Time intervals between sequential FUS
1st−2nd MRI (1st FUS): 12 (3,5–34) weeks (*n* = 45)
2nd−3rd MRI (2nd FUS): 14,5 (6–42) weeks (*n* = 25)
3rd–4th MRI (3rd FUS): 13,5 (8–29) weeks (*n* = 13)
4th−5th MRI (4th FUS): 12 (8–50) weeks (*n* = 8)
5th−6th MRI (5th FUS): 16 (11,5–42) weeks (*n* = 3)
6th−7th MRI (6th FUS): 31 weeks (*n* = 1)

FUS: follow-up study.

### EESC regression

Out of the 95 FUS, there were 66 FUS showing time dependent regression of EESC as expected (24 cases, cancellous to compact bone ratio 8:1); for an example, see Figure 1(b, c). 36/66 FUS were late ones obtained after >24 weeks of treatment. In 17 cases, sequential FUS revealed continuously regressive EESC until near normalization (equivalent to ‘healing’ active-stage Charcot foot): in 9 grade 0 cases after 13(6–34) weeks, and in 8 grade 1 cases after 23(13–70) weeks of continuously regressive EESC (Mann–Whitney U test *p* = 0.034), corresponding to an estimated duration of TCC treatment until ‘healing’ of about 25 weeks (grade 0) versus 35 weeks (grade 1).

### EESC non-regression

There were in total 29 FUS (21 cases, 9 male and 10 female patients) showing progressing, stagnant, or extending EESC, see Table 4. The cancellous to compact bone ratio was 10:1.

**Table 4.** Cases and follow-up studies with regression of bone marrow EESC.

Regression of bone marrow EESC			
	Yes	No^a^	Total
Total cases, n	24	21	45
Cases with grade 0, *n*	9	8	17
Cases with grade 1, *n*	15	13	28
Cases related to renal failure^b^	3	6	9
Total (1st to 6th) FUS, n	66	29	95
1st FUS, *n*	30	15	45
2nd FUS, *n*	17	8	25
3rd FUS, *n*	9	4	13
4th to 6th FUS, *n*	10	2	12

^a^progressing, migrating, or stagnant EESC on isolated follow-up studies (FUS).

^b^in patients with preterminal kidney failure, kidney-transplantation or kidney-pancreas-transplantation.

EESC = edema equivalent signal changes.

### *EESC regression* versus *non-regression*

The proportions of FUS showing EESC regression was independent of the active-stage Charcot foot severity grade, renal failure, and order of the FUS (1st versus 2nd to 6th FUS); all chi^2^ *p* > 0.05. The respective absolute figures are presented in Table 4.

The 29 FUS with non-regressive EESC were assessed individually in relation to confounding factors, whereby 5 hypothetical clusters emerged.

### Cluster I: false early worsening of EESC

In one grade 0 and one grade 1 case, the 1st FUS displayed more EESC than the diagnostic MRI study acquired 11 and 14.5 weeks before. Diagnostic MRI had been performed only 4–9 days after symptom onset, when the acute inflammatory response of the bones probably was still incomplete. Soft-tissue swelling, however, had improved at the time of the 1st FUS.

### Cluster II: true early worsening of EESC

In 4 cases, effective TCC treatment had been unduly withheld for 1.5–6 weeks after the baseline MRI (which had been performed 1.5–7 weeks after symptom onset). The 1^st^ FUS obtained 6–18 weeks after baseline MRI displayed worsened bony EESC, while foot swelling had decreased.

### Cluster III: late worsening after early improvement of EESC (for an example, see Figure 1(d))

In 5 cases, EESC worsening was noted on 6 FUS (1th FUS: *n* = 1, 2nd FUS: *n* = 3, 3rd or 4th FUS: *n* = 2) which was clearly related to trauma from premature reloading and was accompanied by recurrence of swelling and hyperthermia. Further FUS showed improved EESC.

### Cluster IV: migrating EESC

In 6 cases, 6 FUS (1th FUS: *n* = 2, 2nd FUS: *n* = 3, 3rd FUS: *n* = 1) revealed newly formed EESC remote from the initial EESC lesion (which was reduced at the same time). In two grade 1 active Charcot foot cases, EESC had extended from an isolated bone to neighboring ones. In another grade 1 case, a single hit to a furniture with the unshod foot caused a new spot of EESC. In two grade 0 and one grade 1 cases, EESC appeared in a speckled pattern in various bones distant from the initial EESC focus. This migration of bone EESC was not associated with relapse of swelling.

### Cluster V: unchanging/stagnant EESC

In 7 grade 1 cases with multiple macrofractures on baseline MRI, EESC transiently stagnated in 11 FUS (1st FUS: *n* = 6, 2nd FUS *n* = 2, 3rd FUS: *n* = 2, 4rd FUS: *n* = 1) while soft-tissue edema and hyperthermia had slightly decreased. In two cases, serial FUS showed stagnant EESC, while a palpable bone mass was noted and plain x-ray showed hypertrophic callus and fusion of joints and bone fragments.

### Miscellaneous

In three grade 1 cases, EESC was associated with multiple articular fractures and luxations of the Lisfranc joint, and with large (3×3 cm) fluid collections in adjacent fascia and other soft tissues, as shown on baseline diagnostic MRI scans (probably synovial cysts leaking from ruptured joint capsules, and/or fracture hematoma). The fluid collection, which was mistaken by two radiologists as abscess formation, was found reduced in a FUS 4 months later in one case, or unchanged in a hasty FUS 3.5 weeks later in another case. In a third case, such a fluid collection remained constant over 18 weeks, and was noted to be decreased only 17 weeks later (treatment compliance in this case was doubtful). In two further cases, MRI reports noted plantar fasciitis in addition to EESC.

## Discussion

The present study confirms that monitoring the treatment response to TCC of active-stage Charcot foot by conventional MRI is feasible and straight forward, revealing any potential deviation from the physiologic evolution of posttraumatic EESC. From our data, three hypotheses were generated:

(a) EESC resolution in active-stage Charcot foot, that is the recovery of the condition, is consistent with fracture healing [20,28],

(b) non-regression of EESC in FUS is a temporary phenomenon related to various potential causes,

(c) the time to full EESC resolution may be shorter in cases with microtrabecular fracture only (grade 0), than in cases with macrofracture (grade 1).

(a) Recovery of active-stage Charcot foot is consistent with fracture healing, that is healing of closed metaphyseal, diaphyseal, articular, and/or intraosseous fracture (‘bone bruise’ [28,29]). Water-like MRI lesions feature both, acute focal inflammation with microvascular hyperpermeability (in bone and in uninjured adjoining soft tissues, due to inflammatory cytokines [30]), and chronic focal hyperemia (from newly grown fibrovascular reparative tissue preceding woven bone callus) [30–32]. Histopathology of active-stage Charcot foot has revealed all of these entities [33,34] as part of the physiologic secondary healing of cortical and cancellous bone fractures [27,30], and of reactive adjoining soft-tissue inflammation like tenosynovitis [35,36].

According to two previous short-term MRI studies, the full extent of EESC takes about 2–3 weeks to develop in cancellous bone fracture, and 3–6 weeks in compact bone fracture [12,13]. In this early posttraumatic phase, EESC is indicative of acute destructive inflammatory processes at the site of the injury (equivalent to osteolysis on plain x-ray). The accompanying high signal T2w changes in soft tissue decrease already early (within 4 weeks) after onset of treatment, consistent with subsiding inflammation and clinical improvement. By contrast, EESC decreases gradually over several months (4–7 months [20,28,29]), consistent with the gradual transformation of newly grown fibrovascular tissue into mineralized woven bone callus (hard callus). Hard callus appears as osteosclerosis only on plain x-ray (or CT-scan). Hence, interpretation of EESC may require (i) assessment of soft-tissue signal intensity, and (ii) simultaneous x-ray in order to differentiate between early destructive inflammatory processes and later reparative callus formation (particularly in grade 1 cases with macrofractures [20,30]), together with (iii) monitoring serum markers of fracture healing [37]. Needless to say that fracture healing (and EESC regression) takes time and, hence, time intervals of less than 3 months are not recommended for regular MRI monitoring of active-stage Charcot foot. Remodeling of lamellar bone from woven bone takes up to several years and does not appear on MRI.

(b) Contrary to earlier claims [38], EESC in the present study did not regress without fluctuation and migration in some cases. Temporary non-regression (or progression) of EESC was observed in the present study in about 1/3 of all FUS. A similar quota was found by Wikeroy et al. assessing the recovery of bone bruise in the traumatized hip [39]. Non-regression of EESC in active-stage Charcot foot could mean either normal or disturbed healing processes, or lack or proof of a treatment effect, respectively. Therefore, any non-regression of EESC under TCC treatment needs to be explored carefully, accounting amongst others for treatment circumstances. In the present study, non-regression of EESC was unrelated to cancellous versus compact bone, kidney function status, order of FUS, or active-stage Charcot foot severity grade (0 versus 1), respectively. However, concomitant clinical features suggested several potential causes for this phenomenon. Premature re-loading was clearly causative for active-stage Charcot foot relapse in five cases, and withholding offloading and immobilizing treatment was responsible for non-regression (or progression) of EESC in another four cases. Unchanged or stagnant EESC on eleven FUS (in grade 1 cases) was attributed to impaired healing associated with hypertrophic fibrovascular tissue and callus, as indicated by osteosclerosis and joint fusion (ankylosis) seen on plain x-rays obtained simultaneously. Most likely, ineffective immobilization of the bone fragments was the underlying factor, causing hyperplasia of fibrous rather than of mineralized callus [32,40].

In six cases, newly formed EESC appeared in speckled pattern remote from the initial focus of EESC. Previously, Thorning et al. [41] had noticed comparable spotty bone marrow edema in feet with neuropathic foot ulcerations during offloading with appropriate orthoses. This pattern of EESC might not predict future Charcot osteoarthropathy, and is more common in end-stage renal disease, they suggested [41]. However, we were unable to underpin their assumptions by our small study. Such an unspecific and unexplained EESC was also observed in non-diabetic feet after offloading and immobilizing treatment [42].

c)Speed of recovery of active-stage Charcot foot (‘healing’ by disappearance of EESC) seems to differ according to the severity grade existing at onset of treatment, consistent with Arendt and Griffiths [20]. Grade 0 cases, which may heal by direct (primary) fracture healing [30], seemed to require shorter TCC treatment (approximately 25 weeks [28]) until ‘healing’ than grade 1 cases (approximately 35 weeks), which invariably heal by indirect (secondary) fracture healing [30]. In a previous retrospective study based on clinical and x-ray monitoring [25], we found a non-significant difference in average ‘healing’ time of 1 month between grade 0 and 1. Disappearance of EESC seems to require longer treatment than ‘healing’ defined by clinical and plain x-ray criteria [23]. It has to be kept in mind that ‘healing’ of active-stage Charcot foot of either grade will leave behind residual minor or major abnormalities in bones [43], joints or ligaments, and functional deficits (e.g. foot stiffness). In addition, debilitating skeletal deformities like the breakdown of the longitudinal arch [44] are residuals mainly in severe grade 1 cases and can be avoided by early treatment of active-stage Charcot foot grade 0.

Outlook: Standardization of routine MRI interpretation and reporting would be highly desirable for follow-up comparisons. For instance, signal intensity could be reported semiquantitatively, as Krüger et al. have shown in native MRI studies [13]. They measured signal intensity of affected bone or soft tissue in relation to signal intensity of a reference ‘region of interest’. Corresponding to certain features of the fracture healing processes, the calculated ratio changes over time. Other standardization models have been effectively applied [29,45,46]. Signal intensities of bones and adjoining soft tissues could easily be reported according to the grading schemes of Arendt and Griffiths [20] or Kiuru et al. [11] for stress fractures. This would help to perceive clinically meaningful responses to treatment at a glance. To facilitate differentiation between ‘destructive’ EESC (inflammation soon after injury) and ‘reparative’ EESC (from newly formed fibrovascular tissues) in grade 1 cases, supplemental plain x-ray/CT-scan FUS may be needed for interpretation of MR images. Open questions remain as to the best conventional MRI protocol for monitoring the treatment response of active-stage Charcot foot, including the necessity of contrast media application.

### Limitations of the study

The study is the first comprehensive report on the potential effects of Charcot foot healing on MRI morphology, on a relatively large case series. It has, however, considerable deficits due its retrospective nature and explorative design. Based on routine clinical material, the findings could only be described and not analyzed in great detail. The sample size was limited. Only a fraction of the study population was followed-up until ‘healing’. Follow-up intervals and follow-up periods varied between the cases. The study population was heterogeneous. Hence, our observations and conclusions cannot be generalized and need to be supported by controlled prospective studies, one of which is already under way [47].

## Conclusions

Taken together, the present study suggests that temporal changes in posttraumatic EESC in the treated active-stage Charcot foot can be monitored by regular conventional MRI follow-up studies. The changes in EESC need to be interpreted carefully, in relation to the physiologic fluctuations after an acute fracture. Following about 12 weeks of offloading and immobilization treatment by TCC, a regression of EESC can be faithfully expected, compared to an initial MRI obtained by the time of fully developed initial EESC (6–8 weeks after the injury, e.g. after swelling onset). Further study is required to firmly establish the evolution of EESC in the treated active-stage Charcot foot.
